# Effect of hearing ability on inflammation and glymphatic function affecting cognition in older adults

**DOI:** 10.1007/s11357-025-01880-7

**Published:** 2025-09-11

**Authors:** Weijie Ye, Chunhua Xing, Jun Yao, Xiaomin Xu, Zihuai Fang, Xindao Yin, Richard Salvi, Yu-Chen Chen, Yuexin Cai

**Affiliations:** 1 Department of Otolaryngology, Sun Yat-Sen Memorial Hospital, Sun Yat-Sen University, 107 West Yanjiang Road, Guangzhou, 510120 China; 2 Institute of Hearing and Speech-Language Science, Sun Yat-Sen University, 107 West Yanjiang Road, Guangzhou, 510120 China; 3 Department of Radiology, Nanjing First Hospital, Nanjing Medical University, No.68, Changle Road, Nanjing, 210006 China; 4 Center for Hearing and Deafness, University at Buffalo, The State University of New York, Buffalo, NY 14214 USA; 5 Medical Imaging Research Institute, Department of Radiology, Nanjing First Hospital, Nanjing Medical University, No.68, Changle Road, Nanjing, 210006 China

**Keywords:** Age-related hearing loss, Glymphatic system function, Inflammation, Cognition

## Abstract

The mechanisms linking hearing ability, inflammation, glymphatic system function, and cognitive impairment in older adults are largely unknown. To investigate this issues, magnetic resonance imaging (MRI) was used to test for dysfunctions in the glymphatic system of older adults with hearing loss and to determine the relationship of glymphatic dysfunction, inflammation and cognitive deficits. This cross-sectional observational study included participants with ARHL and healthy controls (HCs) between January 2021 and December 2023. Participants underwent MRI scans of the glymphatic indices and clinical assessment of auditory, neuropsychological, and inflammatory measures. Multimodal MRI indices were used as proxies of glymphatic function and compared with measures of inflammation and cognition dysfunction in the older adults with hearing loss and control groups. Mann–Whitney *U* test, Spearman rank correlation and Mediation analysis were conducted. A total of 130 hearing loss patients (mean age years, 64.10 ± 3.43 [SD], 67 males) and 121 healthy controls (mean age years, 63.55 ± 3.49 [SD], 68 males) were included. The hearing loss group differed significantly from normal hearing control on MRI glymphatic measures of choroid plexus volume (CPV) (1.48 vs 1.37, *p* = 0.0004), enlarged perivascular spaces (EPVS) (1.74 vs 1.55, *p* < 0.0001) and diffusion tensor image analysis along the perivascular space (DTI-ALPS) (1.47 vs 1.63, *p* < 0.0001). Hearing loss severity was associated with higher values of CPV and EPVS and lower values of ALPS and strongly correlated with higher levels of inflammation (all *FDR q* < 0.05). Mediation analysis showed that ALPS and CPV mediate the relationship between hearing loss and scores on the Montreal Cognitive Assessment (MoCA) and Digit Symbol Substitution Test (DSST), respectively. Our findings support a potential associative pathway that inflammation and glymphatic dysfunction act as plausible intermediate factors facilitating the pathological relationship linking the increase in hearing loss in older adults to decline in cognition.

**Graphical Abstract:**

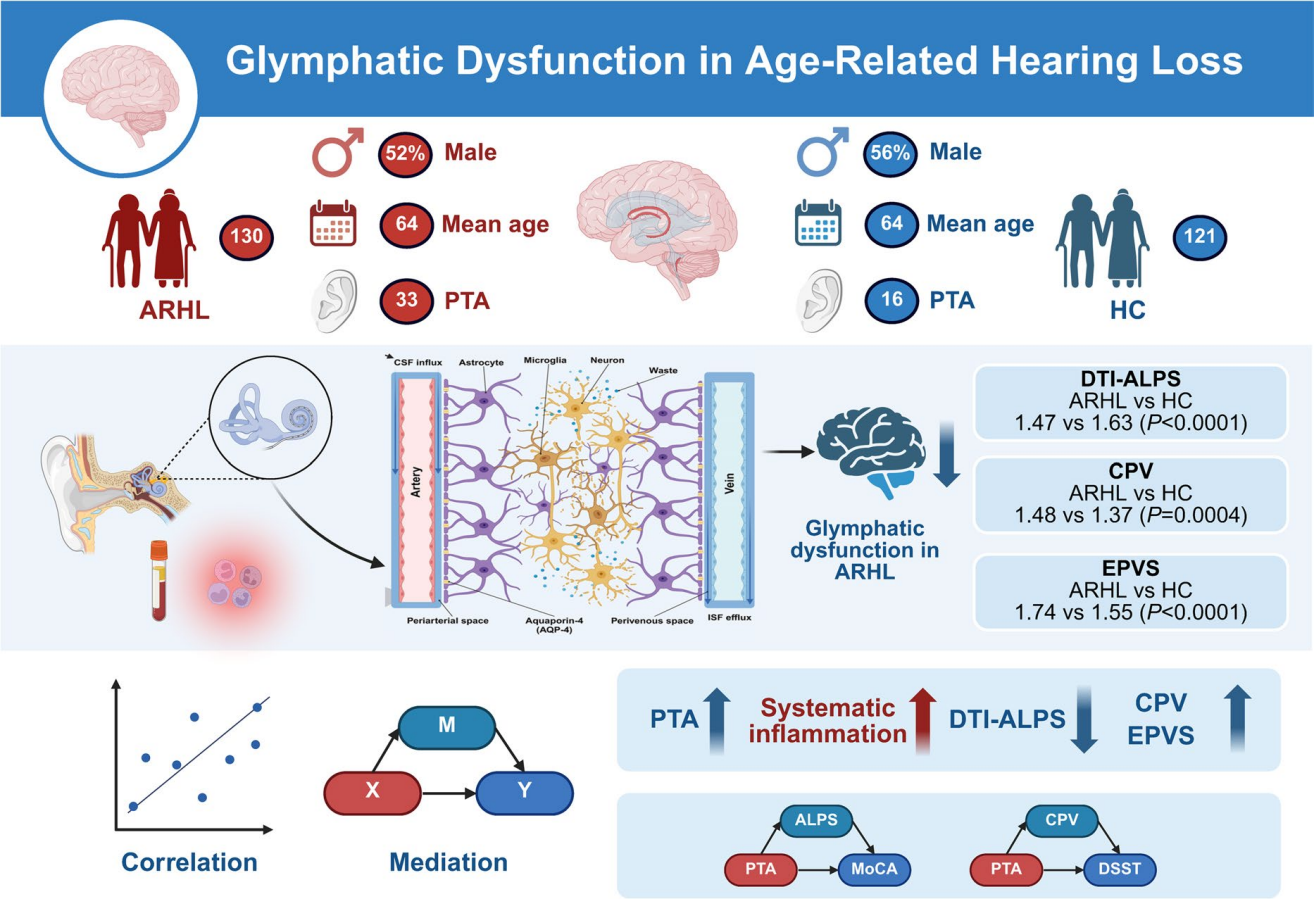

**Supplementary Information:**

The online version contains supplementary material available at 10.1007/s11357-025-01880-7.

## Introduction

Age-related hearing loss (ARHL), the most common sensory deficit associated with aging, is a progressive bilateral sensorineural hearing loss that progresses from high to low frequencies with aging [1, 2]. According to World Report On Hearing released by WHO, over 42% of people with hearing loss are aged 60 or above and the prevalence increases exponentially with age posing significant societal and economic burdens [3]. To add insult to injury, hearing loss is associated with cognitive decline. Moreover, averting ARHL represents the single largest modifiable risk factor for preventing dementia [4, 5].

Diverse mechanisms have been proposed to explain the cognitive function decline in older adults with hearing loss, such as increased cognitive perceptual load, sensory deprivation, information degradation, and social isolation [6]. However, the common cause hypothesis has gained more acceptance based on growing evidence linking cognitive decline with chronic inflammation and neurotransmitter imbalance [7–10]. Prominent among the models is inflamm-aging, an age-related increase in low-grade, sterile chronic inflammation that is putatively responsible for a plethora of age-related diseases such as Alzheimer’s disease, Parkinson’s disease, and ARHL [11–13]. In Alzheimer’s, immune activation of microglia and astrocytes is considered a critical component of disease progression [14–16]. While numerous studies have explored cochlear inflammation as a trigger for ARHL, few have connected it to neuroinflammation, primarily due to the lack of effective in *vivo* methods for assessing generalized neuroinflammation [17].

**Fig. 1:**
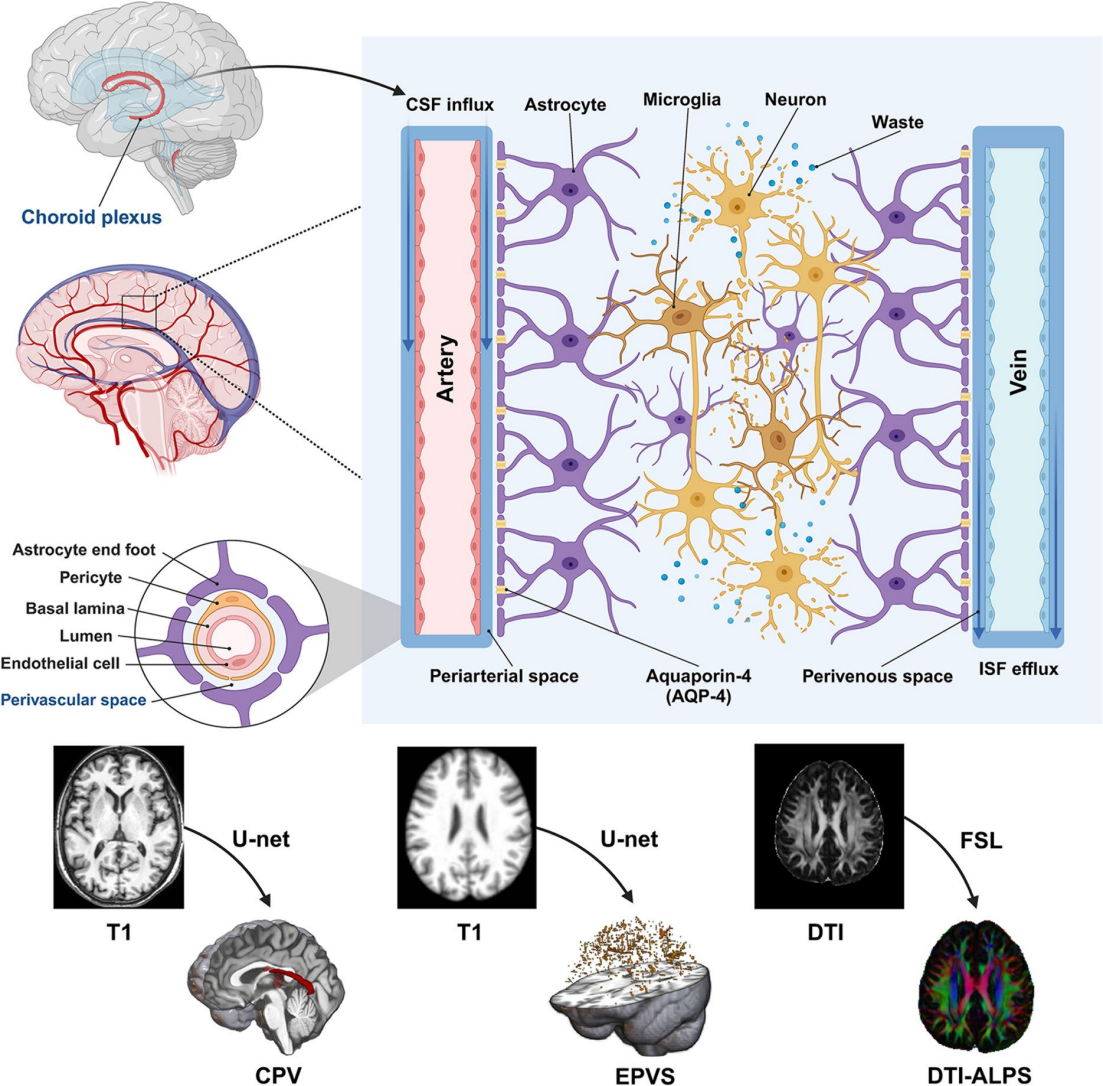
Schematic diagram of the glymphatic system. The initial stage involves generation of cerebrospinal fluid (CSF) by the choroid plexus and inflow of CSF through periarterial spaces. Next stage, CSF flow into brain parenchyma. Soluble waste is washed out by CSF/ISF efflux through perivenous and drains into the peripheral lymphatic vessels in the last stage. Created with BioRender.com

The glymphatic system, a gateway for neuroinflammation [18], is a recently discovered network which plays a pivotal role in maintaining homeostasis and removing metabolic waste from the brain, which may provide previously underexplored link connecting ARHL and cognitive decline [19, 20]. Glymphatic flow involves three stages (Fig. 1). The first stage involves generation of cerebrospinal fluid (CSF) by the choroid plexus and inflow of CSF through periarterial spaces. In the second stage, CSF flows into brain parenchyma through Aquaporin-4 (AQP-4), polarized channel in the end-foot of astrocytes permitting fluid exchange with interstitial fluid (ISF) [21]. Microglia and astrocytes play an essential role regulating neuroinflammation by secreting inflammatory factors such as tumor necrosis factor (TNF)-α, interleukin (IL)−1β, and IL-6) and interacting with each other [22]. In the third stage, soluble waste is washed out by ISF efflux through the perivenous network which drains into the peripheral lymphatic vessels [23].

The glymphatic system is regularly evaluated by proton emission tomography (PET) or gadolinium-based contrast-enhanced MRI; however, several non-invasive MRI indices are validated and useful for indirect glymphatic evaluation [24], such as choroid plexus volume (CPV) [25], enlarged perivascular spaces (EPVS) [26], diffusion tensor image analysis along the perivascular space (DTI-ALPS) [27–29] and free water (FW) [26]. Aberrant glymphatic system function purportedly contributes to pathophysiology of CNS ageing, neurodegenerative diseases, and other brain injuries [30, 31]. ARHL patients with cognitive impairment have lower DTI-ALPS index values than those without cognitive impairment or age-matched healthy controls; the decline in DTI-ALPS index values was significantly correlated with lower scores on global cognitive performance [32]. However, it is unclear whether other components of the glymphatic system undergo changes in ARHL and if increased inflammation contributed to the glymphatic system dysfunction and cognitive decline associated with ARHL.

To determine if glymphatic system dysfunction and inflammation were associated with cognitive decline among individuals with ARHL, we used multimodal MRI to obtain metrics as proxies of glymphatic function and determined their relationships to measure of neuroinflammation, cognitive performance in ARHL. Building on this framework, our study aimed to explore whether peripheral inflammation and glymphatic dysfunction may serve as potential neurobiological links between hearing loss and cognitive impairment in older adults. Based on prior evidence, we proposed that older adults with hearing loss would exhibit disrupted glymphatic function relative to healthy controls. Moreover, we expected that glymphatic dysfunction would be closely associated with hearing ability, systemic inflammation, and cognitive decline. Finally, we aimed to explore whether inflammation and glymphatic alterations could statistically mediate the relationship between hearing loss and cognitive impairment.

## Subjects and methods

### Study participants

This was a cross-sectional observational study conducted at a single clinical center. Approval for the study was obtained from the Research Ethics Committee of our center and the study was conducted in accordance with the Helsinki Declaration. Patients over 60 years old and right-handed with chief complaints of hearing loss were recruited from the Department of Otolaryngology and Department of Radiology between January 2021 and December 2023. Age-matched healthy controls (HCs) were recruited from nearby communities and were matched to cases based on age, sex, and years of education. All subjects provided written informed consent before their participation in the study protocol. Participants were first assessed by otoscopy and tympanometry to confirm normal middle-ear function and eliminate those with conductive hearing loss (e.g., otitis media, cerumen impaction). Participants who passed the initial screening underwent further examination detailed in clinical assessment.

The exclusion criteria included based on clinical records and structured interviews: (1) middle-ear or inner-ear diseases that affected hearing threshold (e.g., otitis media, tinnitus, hyperacusis, Meniere’s disease and otosclerosis); (2) conductive hearing loss (a mean air-bone difference at 0.5, 1, 2, and 4 kHz) > 10 dB in one or both ears; (3) a history of noise exposure or otologic surgery; (4) neurological or mental disorders (e.g., Alzheimer’s disease, Parkinson’s disease, epilepsy, cerebral or acoustic neuroma, head injury, stroke, depression and psychiatric history), major diseases (e.g., anemia, thyroid dysfunction, cancer), smoking or alcohol addiction; (5) poorly controlled hypertension, diabetes, hyperlipidemia, or other systemic diseases, or receiving unstable pharmacologic treatment; (6) recent history of acute inflammation or use of anti-inflammatory drugs; (7) contraindications to MRI (e.g., cardiac pacemakers, metal implants).

### Clinical assessment

#### Auditory assessment

Participants initially included were evaluated by pure tone audiometry (PTA) to assess auditory status and degree of hearing loss [33]. PTA was measured in both ears in an acoustically shielded room by a professional audiologist at 0.25, 0.5, 1, 2, 4 and 8 kHz. Patients with ARHL were classified as those having average hearing thresholds at 0.25, 0.5, 1, 2, 4 and 8 kHz above 20 dB HL in the better hearing ear.

#### Neuropsychological assessment

A series of cognitive assessments were performed on all participants in fixed order in a quiet environment. The Minimum Mental State Examination (MMSE) and the Montreal Cognitive Assessment (MoCA) were used to assess global cognitive function. The Verbal Fluency Test (VFT) was conducted to evaluate language function. The Trail Making Test Part A (TMT-A) and Part B (TMT-B), Digit Symbol Substitution Test (DSST), Digit Span Test (DST) were used to comprehensively evaluate attention, working memory, and executive function. The Auditory Verbal Learning Test (AVLT) and delayed recall after a 5-min interval (AVLTd) were used to assess auditory learning and memory. The Clock Drawing Test (CDT), Complex Figure Test (CFT) and CFT-delayed after 3 min interval (CFTd) were used to evaluate visual-spatial function. These cognitive assessments were conducted using Chinese version of the scales, whose reliability and validity have been validated [34–40]. The original scores were converted to z-scores before further analysis by subtracting the mean of all participants and dividing it by the standard deviation for further analyses.

#### Inflammatory factors measurement

Fasting blood samples from all participants were collected in dipotassium-EDTA glass tubes, centrifuged at 4000 rpm for 5 min, and the plasma was extracted and stored in a refrigerator at − 80 ℃. The TNF-α, IL-1β, and IL-6 were measured with enzyme-linked immunosorbent assay (ELISA) kits (Invitrogen, Thermo Fisher Scientific, Waltham, MA, USA).

### MRI acquisition

The T1-weighted (T1w) MRI scans from all participants were acquired using a 3.0 Tesla MRI scanner (Ingenia, Philips Medical Systems, Netherlands) with an 8-channel receiver array head coil. Diffusion tensor imaging (DTI) was acquired using a 3.0 Tesla MR imaging system (Magnetom Prisma, Siemens Healthcare, Germany) with a 64-channel receiver array head coil. Detailed MRI acquisition parameters are provided in Supplementary Method 1.

### Image analysis

#### Choroid plexus volume measurement

Choroid plexus volume was quantified as a proxy of CSF production and immune signaling activity at the blood–CSF interface. To minimize errors introduced by manual segmentation, we applied an automated segmentation method based on a convolutional neural network (CNN) employing the U-Net architecture, specifically tailored for identifying the choroid plexus on T1-weighted (T1w) MRI scans (code available at: https://github.com/Center-of-Imaging-Biomarker-Development/chp_seg) [41].

This neural network framework has been previously validated across a wide age range in adults and demonstrated superior accuracy compared to conventional segmentation algorithms. The step-by-step segmentation workflow is shown in Supplementary Fig. 1A. In short, each subject’s T1w image was first non-linearly registered to the ICBM-MNI 152 T1-weighted brain template using the Advanced Normalization Tools (ANTs) software suite [42]. The registered image was then processed through the 3D U-Net network, generating a map for the choroid plexus, which was subsequently inversely transformed back into native anatomical space. This transformation yielded an individualized choroid plexus mask. The generated mask was visually inspected and verified by a trained imaging analyst. Following this, the final volume calculation was performed in Python (version 3.7.0) utilizing the SimpleITK package (version 2.1.1.1), which enabled the derivation of choroid plexus volumes for each participant [43]. To account for global brain volume variation among individuals, we additionally measured total intracranial volume (TIV), as well as gray matter (GMV), white matter (WMV), and cerebrospinal fluid volumes (CSFV), using the Computational Anatomy Toolbox (CAT12) within the Statistical Parametric Mapping framework (SPM12). This toolbox, developed by the Departments of Psychiatry and Neurology at Jena University Hospital, Germany, provides a robust platform for Voxel-based morphometry. All measurements were normalized by expressing Volumes as a proportion of TIV multiplied by 1000, thus controlling for inter-individual variability in brain size. Subsequent analyses were based on these normalized metrics.

#### Enlarged perivascular spaces measurement

Whole-brain enlarged perivascular spaces were segmented to estimate fluid accumulation associated with interstitial drainage efficiency. To quantify enlarged perivascular spaces (EPVS), we employed a previously validated deep learning-based segmentation model to generate EPVS probability maps from each subject’s T1w MRI scan [44]. This model, which has shown high efficacy in detecting EPVS in the context of cerebral small vessel disease, provided a reliable estimation of EPVS burden [26]. In alignment with both the original model developers’ recommendations and our manual validation, we applied a threshold probability value of 0.1. This threshold optimized the balance between sensitivity and specificity, allowing the inclusion of subtle EPVS features while avoiding excessive false positives. Individual binary masks were thus created for each subject. Following the same quantification strategy as for choroid plexus Volume, we extracted the raw EPVS Volume and further normalized it by expressing it as a ratio to TIV, multiplied by 1000, to mitigate the influence of brain volume variability. A schematic of this segmentation and quantification process is displayed in Supplementary Fig. 1B.

#### DTI-ALPS index calculation

The analysis of the diffusion tensor imaging–along the perivascular space (DTI-ALPS) index, derived from diffusion tensor imaging, reflects the efficiency of perivascular fluid transport in the brain, serving as a surrogate marker of glymphatic function. It was conducted using diffusion-weighted imaging (DWI) data, processed through the DTIFIT module within the FMRIB Software Library (FSL), developed by the Wellcome Centre for Integrative Neuroimaging, University of Oxford, UK (https://fsl.fmrib.ox.ac.uk/fsl/fslwiki/FSL). Details of the processing pipeline are provided in Supplementary Fig. 1C and referenced from previous research studies [27, 29, 32]. Preprocessing included several standard steps: converting DICOM files into NIFTI format, performing brain extraction, correcting for eddy current-induced distortions, and calculating diffusion tensors. After these steps, we utilized the FSLeyes visualization tool to manually place spherical regions of interest (ROIs) with a 5-mm diameter in bilateral projection fiber tracts, association fiber tracts, and subcortical fiber regions, based on their appearance in the color-coded fractional anisotropy (FA) maps. Within these ROIs, diffusivity values were extracted along the primary diffusion directions (x-, y-, and z-axes). These values were then used to compute the DTI-ALPS index according to the following formula: mean (Dxxassoc, Dxxproj)/mean (Dzzassoc, Dyyproj). All the placements of ROI were reviewed by an experienced radiologist.

### Validation analysis

A validation analysis was performed based on the fact that ARHL is greatest at high frequencies [2]. To investigate glymphatic system function and change of inflammation level in patients with high frequency hearing loss, we regrouped all participants based on the average PTA at 4 kHz and 8 kHz in the better ear. Less than 20 dB HL was considered normal hearing, 20 to < 35 dB HL was considered mild hearing loss, 35 to < 50 dB HL was considered moderate hearing loss, and 50 to < 65 dB HL was considered moderate to severe hearing loss [3]. There were no patients with severe or greater hearing loss among the participants included in this study.

### Statistical analysis

Statistical analyses were performed using SPSS 25.0 (Released 2017, IBM, Armonk, NY, USA) and GraphPad Prism 10.2.0 (GraphPad Software, Boston, Massachusetts, USA, www.graphpad.com). The threshold for statistical significance was *p* < 0.05 or *FDR q* < 0.05.

#### Description and between-group comparisons

The Kolmogorov–Smirnov test was used to test data normality. Continuous variables were presented as mean values (SD). Student’s *t* tests were performed to compare the differences in normally distributed continuous data and Mann–Whitney *U* tests were used to compare non-normally distributed data. False discovery rate (FDR) (Benjamini–Hochberg procedure) correction was carried out based on the overall number of pairwise comparisons of cognitive performance. The chi-square test was performed to test for differences in categorical metrics. In validation analyses, the Kruskal–Wallis test was used for comparisons between groups, and the Dunn’s multiple comparisons test was used for pairwise post hoc analyses.

#### Correlation analysis

Spearman rank correlations were performed to assess the relationship between each pair of variables. We performed a series of partial Spearman rank correlation analyses to examine the association between hearing, MRI indices, inflammatory factors, and cognitive performance measures, with age, gender, and education as covariates. The Benjamini–Hochberg FDR method was used to correct for all correlation analyses at the level of q < 0.05. In addition, we performed canonical correlation analyses to reveal potential relationships, a method considered useful in identifying the links between variable sets from different modalities [45, 46]. We explored the relationship between three sets of variables, glymphatic indices, inflammatory factors, and cognitive performance measures.

#### Mediation analysis

Based on significant correlations in the above correlation analyses and the common cause hypothesis linking cognitive decline with chronic inflammation in ARHL, Mediation analysis was conducted using the PROCESS macro (Model 4) with 5000 bootstrap samples [47] was applied to explore the role of inflammation and glymphatic system function in the association between hearing ability and cognitive function. The directionality was specified based on prior evidence indicating that hearing loss contributes to systemic inflammation and cognitive decline. Statistical significance was defined as a *p* < 0.05 or 95% confidence interval excluding zero.

## Results

### The demographic and clinical features

**Fig. 2:**
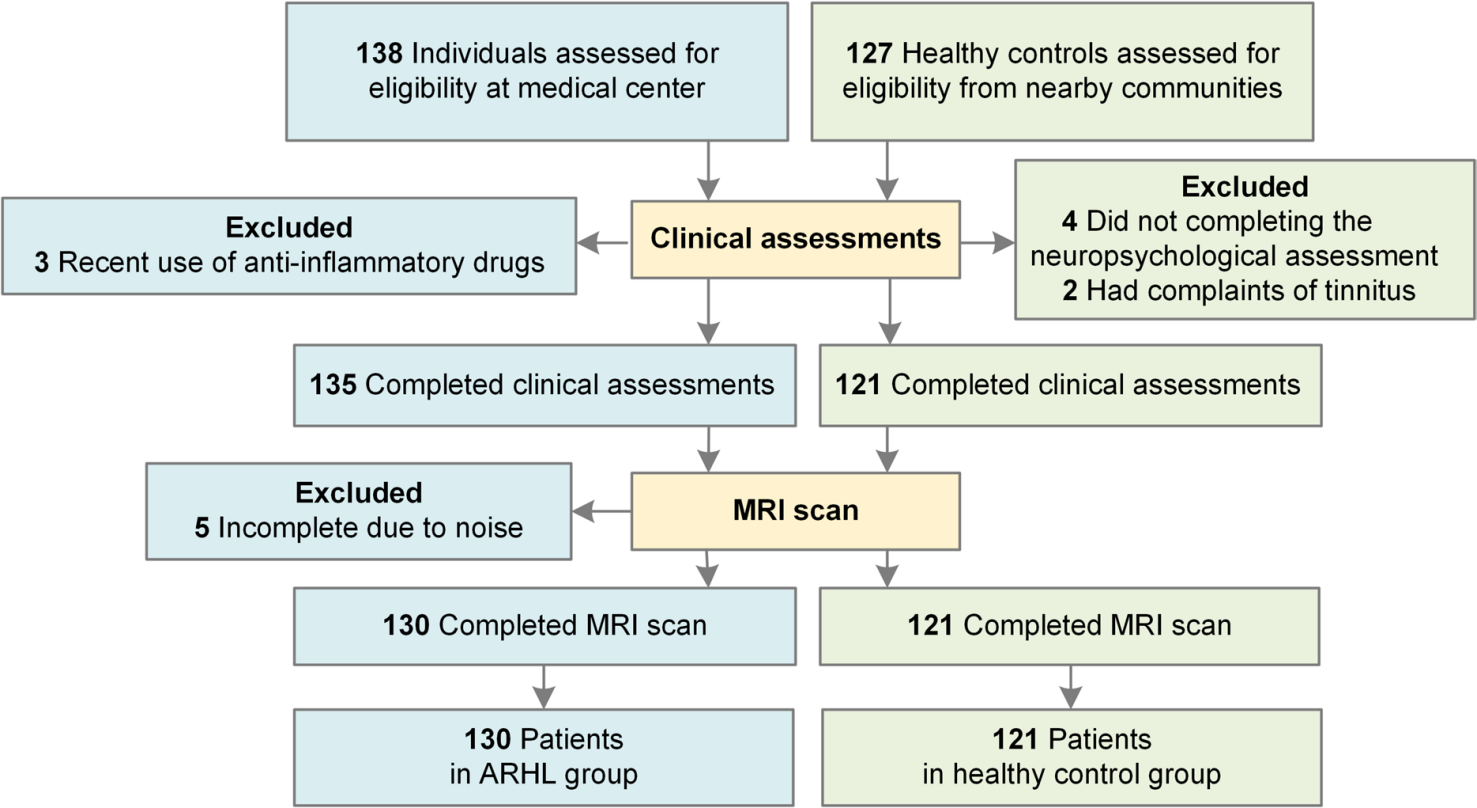
Flow diagram of the participant enrollment process

**Fig. 3:**
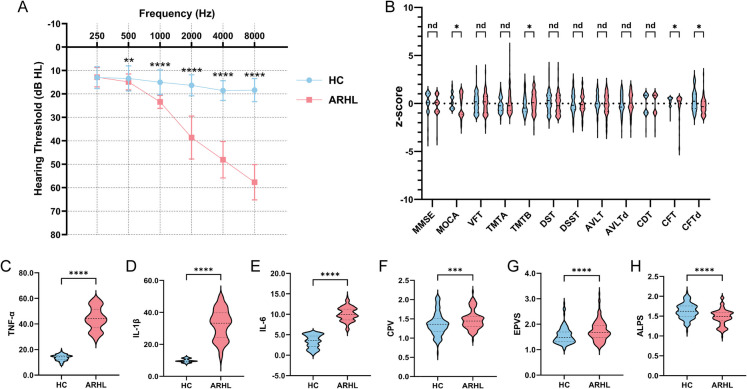
Between-group comparisons. (**A**) Group comparison of average Binaural PTA in the ARHL and HC groups at 0.25, 0.5, 1, 2, 4, and 8 kHz. **B** Neuropsychological assessments were compared between groups using z-score and FDR correction for multiple comparisons. Between-group comparisons of TNF-α (**C**), IL-1β (**D**), IL-6 (**E**), CPV (**F**), EPVS (**G**), and DTI-ALPS (**H**). CPV and EPVS were compared using ratio of total intracranial volume*1000. Abbreviations: ARHL, age-related hearing loss; HC, healthy control; PTA, pure tone audiometry; MMSE, Minimum Mental State Examination; MoCA, Montreal Cognitive Assessment; VFT, Verbal Fluency Test; TMT-A, Trail Making Test-Part A; TMT-B, Trail Making Test-Part B; DSST, Digit Symbol Substitution Test; DST, Digit Span Test; AVLT, Auditory Verbal Learning Test; AVLTd, Auditory Verbal Learning Test-delayed; CDT, Clock Drawing Test; CFT, Complex Figure Test; CFTd, Complex Figure Test-delayed; TNF-α, tumor necrosis factor; IL-1β, interleukin-1β; IL-6, interleukin-6; CPV, choroid plexus volume; EPVS, enlarged perivascular space; DTI-ALPS, diffusion tensor image analysis along the perivascular space, also abbreviated as ALPS. The asterisks (*, **, ***, and ****) indicate statistical significance at *p* < 0.05, *p* < 0.01, *p* < 0.001, and *p* < 0.0001, respectively

Initially, a total of 138 participants with chief complaints of hearing loss and 127 HCs were recruited after screening. Three of ARHLs were excluded due to recent use of anti-inflammatory drugs and five were excluded for inability to tolerate noise during MRI scanning. Among the 127 HCs, four were excluded for not completing the neuropsychological assessment and two were excluded for complaints of tinnitus then transferred for further consultation and treatment. Ultimately, 130 ARHLs (mean age years: 64.10, SD: 3.43; 63 females, 67 males) and 121 HCs (mean age years: 63.55, SD: 3.49; 53 females, 68 males) were included in the study (Fig. 2). Mean PTA of both ears was significantly higher in the ARHL group than in the HC group (*p* < 0.0001). The ARHL group had higher thresholds than HC at 0.5, 1, 2, 4, and 8 kHz (Fig. 3A). Detailed demographic and clinical characteristics of the participants are shown in Table 1.

**Table 1:** Demographic and characteristics of participants

Characteristic	HC (*n* = 121)	ARHL (*n* = 130)	Test statistic	*p* value	*FDR q*
Age, years	63.55 (3.49)	64.10 (3.43)	− 1.491	0.136^a^	-
Gender, F/M	53/68	63/67	0.547	0.459^b^	-
Education, years	11.31 (1.98)	11.68 (2.19)	− 0.892	0.372^a^	-
PTA of left ear, dB HL	15.48 (2.56)	32.73 (4.19)	− 13.702	** < 0.0001**^**a**^	-
PTA of right ear, dB HL	16.05 (3.05)	32.89 (6.42)	− 13.578	** < 0.0001**^**a**^	-
Mean PTA of both ear, dB HL	15.76 (2.02)	32.81 (3.81)	− 13.689	** < 0.0001**^**a**^	-
Mean PTA at 8 kHz, dB HL	18.35 (4.92)	57.63 (7.49)	− 13.727	** < 0.0001**^**a**^	-
Neuropsychological assessments					
MMSE	29.02 (1.14)	28.82 (0.99)	− 2.311	**0.021**^**a**^	0.0501
MoCA	27.28 (1.25)	26.55 (1.97)	− 3.253	**0.001**^**a**^	**0.0069**
VFT	14.47 (3.30)	14.31 (3.74)	− 0.459	0.646^a^	0.7054
TMT-A	66.05 (16.77)	72.60 (26.86)	− 1.142	0.253^a^	0.4348
TMT-B	177.37 (52.05)	193.88 (55.69)	− 2.543	**0.011**^**a**^	**0.0331**
DSST	69.69 (9.01)	69.41 (8.37)	− 0.127	0.899^a^	0.8993
DST	11.58 (1.84)	11.31 (1.82)	− 1.223	0.221^a^	0.4348
AVLT	35.69 (6.71)	34.80 (7.78)	− 0.690	0.490^a^	0.5892
AVLTd	6.79 (2.40)	7.00 (2.39)	− 0.762	0.446^a^	0.5892
CDT	3.50 (0.55)	3.55 (0.54)	− 0.876	0.381^a^	0.5721
CFT	34.45 (1.77)	32.19 (6.03)	− 2.689	**0.007**^**a**^	**0.0288**
CFTd	21.34 (5.66)	18.23 (4.49)	− 4.511	** < 0.0001**^**a**^	** < 0.0001**
Inflammatory factors					
TNF-α	13.75 (2.34)	44.16 (8.84)	− 13.687	** < 0.0001**^**a**^	-
IL-1β	9.85 (1.21)	32.45 (9.43)	− 13.624	** < 0.0001**^**a**^	-
IL-6	3.35 (1.59)	10.10 (1.67)	− 13.687	** < 0.0001**^**a**^	-

*F* female, *M* male, *PTA* pure tone audiometry, *MMSE* Minimum Mental State Examination, *MoCA* Montreal Cognitive Assessment, *VFT* Verbal Fluency Test, *TMT-A* Trail Making Test-Part A, *TMT-B* Trail Making Test-Part B, *DSST* Digit Symbol Substitution Test, *DST* Digit Span Test, *AVLT* Auditory Verbal Learning Test, *AVLTd* Auditory Verbal Learning Test-delayed, *CDT* Clock Drawing Test, *CFT* Complex Figure Test, *CFTd* Complex Figure Test-delayed, *TNF-α* tumor necrosis factor, *IL-1β* interleukin-1β, *IL-6* interleukin-6

Values are presented as mean (1SD). FDR *q*, corrected for multiple comparisons using Benjamini–Hochberg method

^a^Mann–Whitney *U*-test

^b^Chi-square test

Bold values represent significant differences

### Group differences in hearing, inflammation, cognition, and glymphatic markers

The ARHL exhibited worse performance than HC in some assessments (For details, see Table 1 and Fig. 3B). On the cognitive performance tasks, the ARHL group scored significantly lower than HC on the MoCA test (*FDR q* = 0.0069); spent more time than HC on the Trail Making Test-Part B (TMT-B) (*FDR q* = 0.0331). In the CFT and CFTd, the ARHL group scored significantly lower than the HC group respectively (*FDR q* = 0.0288; *FDR q* < 0.0001). Notably, the decline in attention and executive function aligns with domains previously shown to be sensitive to auditory deprivation.

Between-group comparisons of inflammatory factors showed that the levels of inflammatory markers TNF-α (Fig. 3C, *p* < 0.0001), IL-1β (Fig. 3D, *p* < 0.0001) and IL-6 (Fig. 3E, *p* < 0.0001) were significantly higher in the ARHL group than in HC group.

We compared glymphatic MRI indices (CPV, EPVS, ALPS) between the ARHL and control groups. Between group comparisons of MRI indices of glymphatic function showed that the ARHL group exhibited significantly higher CPV values than the HC group (Fig. 3F, *p* = 0.0004) and significantly higher EPVS values than the HC group (Fig. 3G, *p* < 0.0001). The enlargements of CPV and PVS suggest disrupted CSF homeostasis and interstitial fluid clearance, both implicated in neurodegeneration. Additionally, the ARHL group showed significantly lower DTI-ALPS index values than the HC group (Fig. 3H, *p* < 0.0001). This reduction may reflect impaired glymphatic transport capacity, potentially contributing to neurotoxic accumulation. Detailed MRI indices of the participants are shown in Table 2.

**Table 2:** MRI indices of participants

Characteristic	HC (*n* = 121)	ARHL (*n* = 130)	Test statistic	*p* value
TIV, cm^3^	1358.20 (144.90)	1372.35 (153.85)	− 0.749	0.455^b^
GMV, cm^3^	583.41 (49.85)	572.83 (60.91)	1.500	0.135^b^
WMV, cm^3^	497.86 (52.62)	493.71 (64.09)	0.559	0.577^b^
CSF, cm^3^	276.93 (74.45)	305.81 (85.52)	− 2.784	**0.005**^**a**^
CPV, cm^3^	1.85 (0.44)	2.03 (0.42)	− 3.009	**0.003**^**a**^
CPV, ratio of TIV*10^3^	1.37 (0.29)	1.48 (0.24)	− 3.515	**0.0004**^**a**^
EPVS, cm^3^	2.10 (0.50)	2.37 (0.55)	− 5.268	** < 0.0001**^**a**^
EPVS, ratio of TIV*10^3^	1.55 (0.32)	1.74 (0.39)	− 4.355	** < 0.0001**^**a**^
DTI-ALPS	1.63 (0.19)	1.47 (0.20)	− 5.815	** < 0.0001**^**a**^

*TIV* total intracranial volume, *GMV* gray matter volume, *WMV* white matter volume, *CSF* cerebrospinal fluid, *CPV* choroid plexus volume, *EPVS* enlarged perivascular space, *DTI-ALPS* diffusion tensor image analysis along the perivascular space, also abbreviated as ALPS

Values are presented as mean (1SD)

^a^Mann–Whitney *U*-test

^b^Independent two-sample *t*-test

Bold values represent significant differences

### Correlation among hearing ability, MRI markers of glymphatic function, inflammation and cognitive performance

**Fig. 4:**
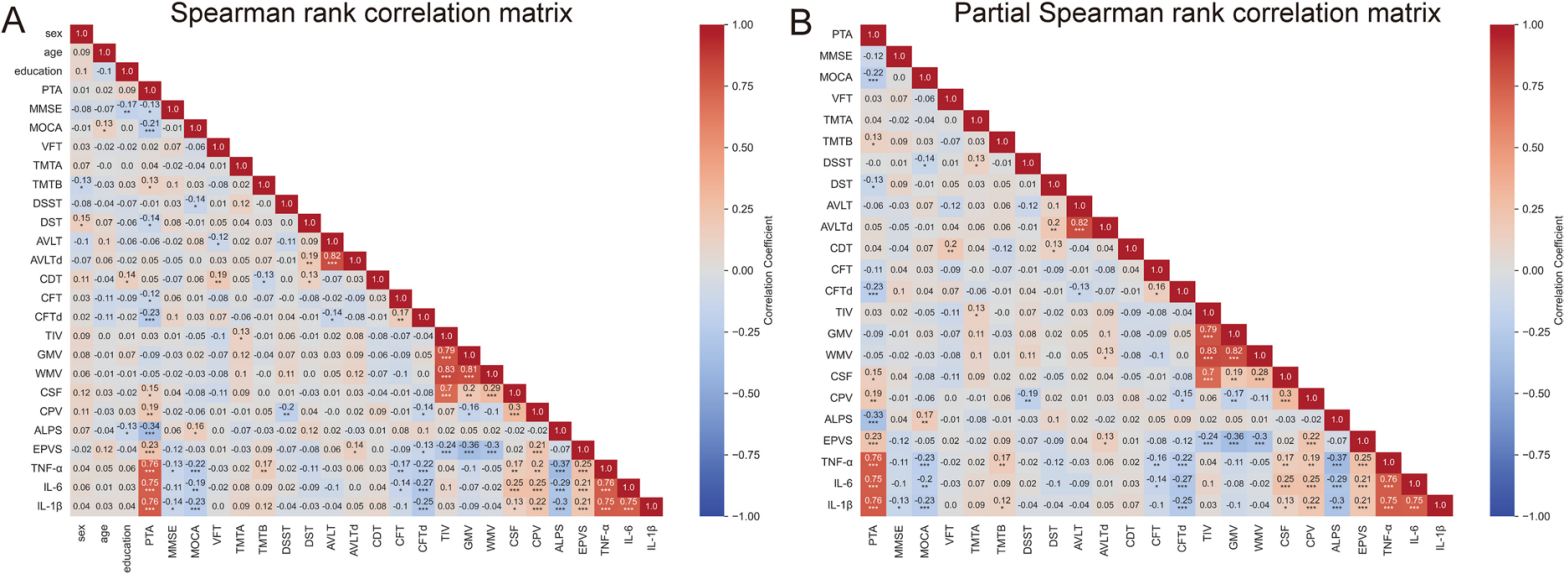
Correlation analyses presented as heatmaps. **A** Correlation heatmaps of all variables using Spearman rank correlation. **B** Correlation heatmaps of variables using partial Spearman rank correlation adding age, gender, and education as covariates. The correlation coefficient *r* is given in the heatmaps and the asterisks (*, **, and ***) indicate statistical significance at *p* < 0.05, *p* < 0.01, and *p* < 0.001, respectively

We examined correlations between glymphatic dysfunction, hearing loss severity, inflammatory markers, and cognitive test performance. Among all participants, age, gender, and education were controlled as covariates to eliminate these variables as potential confounds. Correlation analysis showed that the mean PTA (0.25–8 kHz) of participants was positively correlated with two MRI indices of glymphatic function, CPV (*r* = 0.19, *FDR q* = 0.014) and EPVS (*r* = 0.23, *FDR q* = 0.002), and negatively correlated with ALPS (*r* = − 0.33, *FDR q* < 0.0001). Among the inflammatory markers, higher levels of TNF-α (*r* = 0.76, *FDR q* < 0.0001), IL-1β (*r* = 0.75, *FDR q* < 0.0001), and IL-6 (*r* = 0.76, *FDR q* < 0.0001) were significantly correlated with higher mean PTA values. Additionally, a higher level of TNF-α, IL-1β, and IL-6 were significantly associated with higher CPV and EPVS values, but lower ALPS values. For the relationship between glymphatic function and cognitive performance, ALPS values were positively correlated with MoCA (*r* = 0.17, *FDR q* = 0.034) and CPV values were negatively correlated with DSST (*r* = −0.19, *FDR q* = 0.015) (Fig. 4 and Supplementary Table 1). Hearing loss was associated with reduced ALPS index and increased CPV and PVS volumes, suggesting impaired glymphatic clearance, and also associated with higher level of inflammation and worse cognitive performances.

**Fig. 5:**
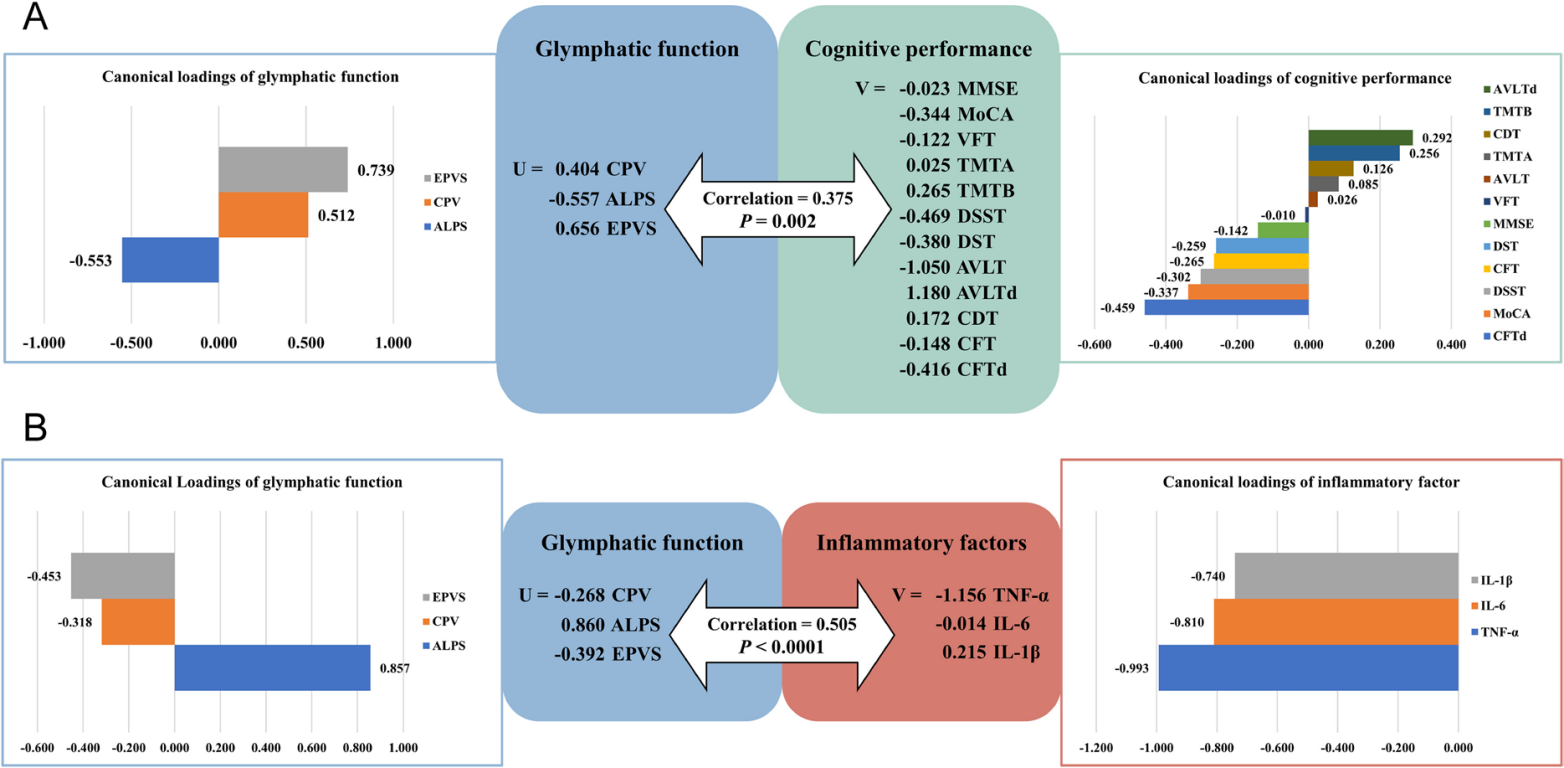
Canonical Correlation Analysis (CCA) to capture interactions between glymphatic function, inflammation and cognition. **A** The canonical correlation analysis describes the correlation between a set of variables that represent glymphatic function and cognitive performance. **B** The canonical correlation analysis describes the correlation between a set of variables that represent glymphatic function and inflammatory factors

Figure 5 showed the canonical correlation analysis aimed at capturing the interactions among glymphatic function, inflammation and cognition, by maximizing the correlation coefficient between two groups by calculating the linear combination of variables in each group. Figure 5A shows the canonical correlation analysis describing the relationship between a set of variables that represent glymphatic function and cognitive performance. The overall canonical correlation coefficient of indices of glymphatic function with cognitive performance was 0.375 (*p* = 0.002). Canonical loadings show the relationship between each glymphatic variable and the linear combination of cognitive performance measures it is associated with. EPVS (U = 0.656) and CPV (U = 0.404) were positively correlated with most cognitive performance scores whereas ALPS (U = − 0.557) was negatively correlated with most cognitive performance scores.

Figure 5B showed the canonical correlation analysis describing the correlation between a set of variables that represent glymphatic function and inflammatory factors. The overall canonical correlation coefficient of indices of glymphatic function with inflammatory factors was 0.505 (*p* < 0.0001). EPVS (U = − 0.392) and CPV (U = − 0.268) indices were negatively correlated with most cognitive performance scores whereas ALPS (U = 0.860) was positively correlated with most cognitive performance scores.

### Inflammatory factors as statistically supported mediators between hearing ability and glymphatic function

**Fig. 6:**
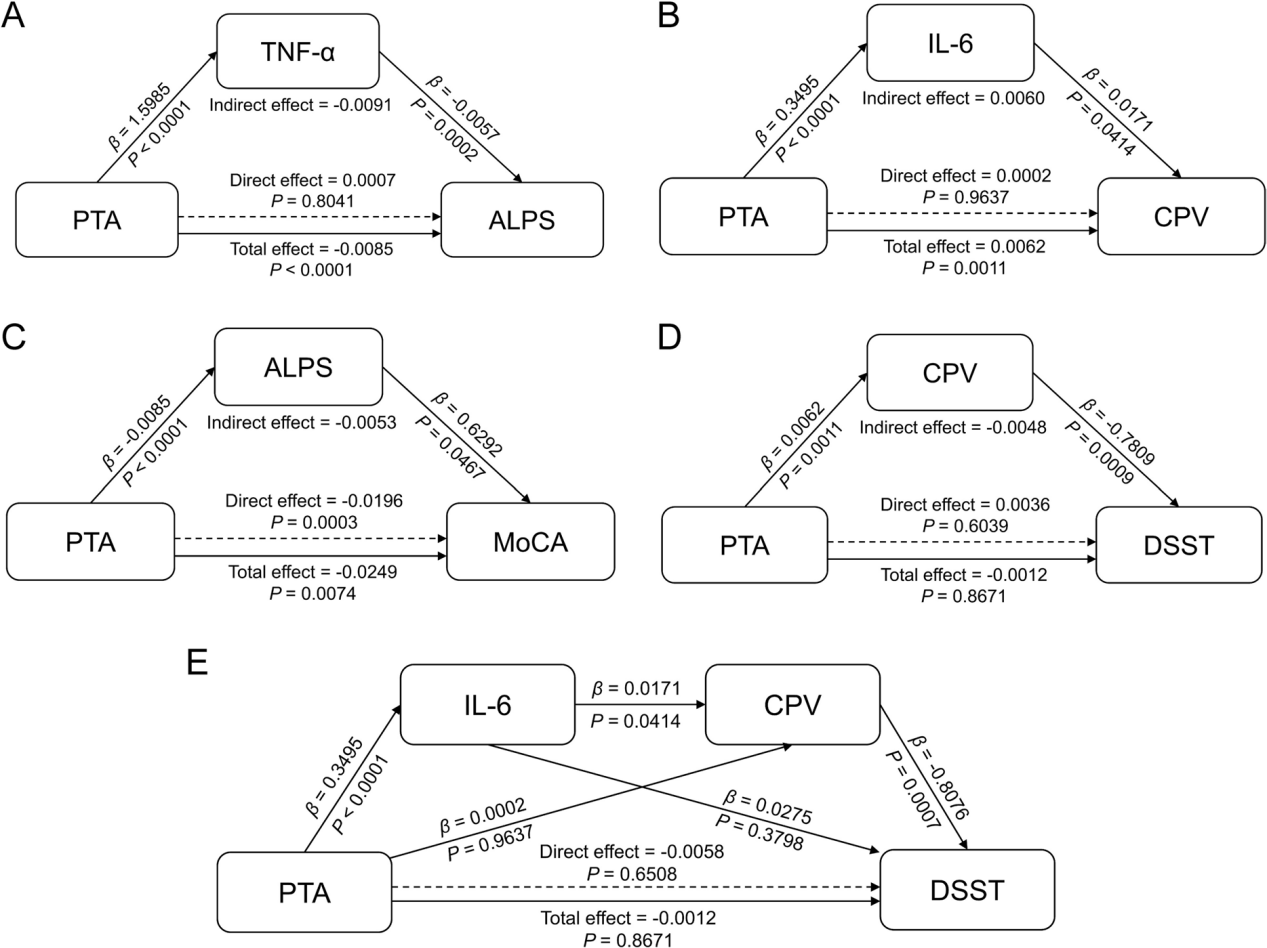
Mediation Analysis of glymphatic function and inflammatory factors. Simple mediation analysis revealed that (**A**) TNF-α as a mediator between PTA and ALPS. **B** IL-6 as a mediator between PTA and CPV. **C** ALPS as a mediator between PTA and MoCA. **D** CPV as a mediator between PTA and DSST. **E** Serial mediation analysis demonstrated that IL-6 and CPV mediated the relationship between PTA and DSST. Abbreviations: PTA, pure tone audiometry; MoCA, Montreal Cognitive Assessment; DSST, Digit Symbol Substitution Test; TNF-α, tumor necrosis factor; IL-6, interleukin-6; CPV, choroid plexus volume; DTI-ALPS, diffusion tensor image analysis along the perivascular space, also abbreviated as ALPS

Based on the correlation analyses, we speculated that inflammation mediated the relationship between PTA and the glymphatic system. We performed mediation analyses to determine whether inflammation mediate the relationship between hearing loss and glymphatic dysfunction. The simple mediation analysis of glymphatic function and inflammatory factors is shown in Fig. 6A to B. TNF-α showed significant mediation effects between PTA and ALPS (indirect effect = − 0.0091, 95% confidential interval [CI] = − 0.0144 to − 0.0040) (Fig. 6A). IL-6 showed significant mediation effects between PTA and CPV (indirect effect = 0.0060, 95% CI = 0.0001 to 0.0117) (Fig. 6B). Details of the simple mediation analysis are shown in Supplementary Table 2.

### Glymphatic function as statistically supported mediators between hearing ability and cognitive performance

We also performed mediation analyses to determine whether glymphatic dysfunction mediate the relationship between hearing loss and cognitive decline. Figure 6C to D showed the simple mediation analysis between glymphatic function and cognitive factors. ALPS showed a significant mediation effect between PTA and MoCA (indirect effect = − 0.0053, 95% CI = − 0.0115 to − 0.0001) (Fig. 6C). CPV showed a significant mediation effect between PTA and DSST (indirect effect = − 0.0048, 95% CI = − 0.0094 to − 0.0013) (Fig. 6D). Details of the simple mediation analysis are shown in Supplementary Table 3.

In addition, we conducted a serial mediation analysis to explore the pathway linking PTA, inflammation and cognitive performance based on the above simple mediation analyses. The pathways from PTA to IL-6, IL-6 to CPV, and CPV to DSST showed a significant mediating effect (indirect effect = − 0.0048, 95% CI = − 0.0109 to − 0.0002) (Fig. 6E, details in Supplementary Table 4).

### With the increase in high-frequency hearing loss, worse glymphatic function, higher inflammation levels, worse cognitive performance

Based on the average PTA at 4 kHz and 8 kHz in the better ear, 77 participants were grouped into normal hearing (mean: 63.66 years; SD 3.87; 32 females, 45 males), 54 into mild hearing loss (mean: 64.0 years, SD 3.50; 28 females, 26 males), 48 into moderate hearing loss (mean: 64.67 years, SD 3.20; 23 females, 25 males), and 72 into moderate to severe hearing loss (mean: 63.33 years, SD: 3.09; 33 females, 39 males). There were no significant differences in age and sex among the groups. Details of between-group comparisons are presented in Supplementary Table 5. Briefly, MMSE (*p* = 0.050), MoCA (*p* = 0.003), CFTd (*p* < 0.0001), inflammatory factors, and MRI indices of glymphatic function differed significantly among groups (see detailed pairwise comparisons between multiple groups in Supplementary Fig. 2).

## Discussion

In this study, we utilized multimodal MRI images to comprehensively evaluate the glymphatic system function in older adults with hearing loss, including segmentation of CPV and EPVS utilizing T1w and calculation of ALPS index using DTI and to investigate their relationships with hearing ability, systematic inflammation, and cognitive performance in older adults. Based on our hypotheses, our study found that glymphatic function in ARHL disrupted compared to healthy controls and also observed robust correlations among hearing ability, inflammation levels, glymphatic dysfunction, and cognitive decline. Moreover, mediation analyses statistically revealed that inflammation and glymphatic dysfunction partially mediated the association between hearing loss and cognitive impairment. These findings unravel that “hearing loss—inflammation—glymphatic dysfunction—cognition” could be a new perspective to understand the underlying mechanism between hearing loss and cognitive decline in the elderly. Therefore, maintaining the stability of glymphatic system function plays a crucial role in improving cognitive function in older adults with hearing loss.

### Glymphatic dysfunction as potential mechanisms linking hearing loss and cognition

Consistent with our findings, previous studies have also demonstrated glymphatic system dysfunction in congenital sensorineural hearing loss (CSNHL) and a negative correlation between the DTI-ALPS index and age in children with CSNHL [48]. Meanwhile, an animal study demonstrated that cerebrospinal fluid transport could comprise an accessible route for gene delivery to the adult inner ear, based on the fact that a bony channel called the cochlear aqueduct connecting the cerebrospinal fluid and the fluid of the inner ear [49]. Following this line of thought, it is reasonable to speculate that ear-brain glymphatic system could offer a new perspective on how auditory health impacts neurological function [50].

The glymphatic system, regarded as a bridge between systemic inflammation and neuroinflammation, has been shown to be impaired in numerous neurodegenerative diseases including age-related diseases, mild cognitive impairment, and Alzheimer’s disease [51]. These findings suggest that glymphatic dysfunction may underlie the link between hearing loss and cognitive impairment in older adults.

### Multimodal MRI techniques for glymphatic function assessment in older adults with hearing loss

Compared to conventional gadolinium contrast MRI imaging, our multimodal MRI analyses effectively and non-invasively provided specific, detailed and clinically relevant information regarding the functional status of the glymphatic system. These metrics could prove useful in evaluating the role of the glymphatic system in other hearing related disorders associated with cognitive decline and other neural comorbidities [52–57].

The choroid plexus, regarded as a unique neuro-immunological interface, not only produces cerebrospinal fluid, but also integrates signals from the central nervous system parenchyma with signals from circulating immune cells, and selectively recruits peripheral leukocytes to the central nervous system parenchyma [58]. In Alzheimer’s disease, increase in CPV was associated with greater Aβ plaque formation and poorer cognitive function [25]. EPVS volume is a major indicator of the increase in periarterial space, representing the inflow of CSF into the brain parenchyma [59]. Enlargement of the PVS of patients with carotid plaque disease is associated with cognitive impairment [60, 61]. The ALPS index, which measures the spatial diffusion around the deep medullary vein, mainly reflects the outflow of CSF/ISF [62]. Factors that aggravate neuroinflammation may exacerbate disease progression partly by damaging the glymphatic system essential for clearing pathogenic protein, such as beta-amyloid [19]. Mild traumatic brain injury leads to disruptions in the glymphatic system and sleep, which are associated with lower ALPS values and cognitive impairment [63, 64]. In our study, we compared these indices in the HC and ARHL groups and observed major differences suggesting that the glymphatic system is significantly impaired in individuals with ARHL. Moreover, the ARHL glymphatic dysfunctions are correlated with inflammatory and cognitive performance deficits.

Older adults with hearing loss showed higher CPV than HCs for reasons that are poorly understood. The choroid plexus not only plays a pivotal role in CSF production but is also essential for regulating the transfer of immune cells from the brain parenchyma into CSF, a proposed marker of neuroinflammation [65]. Our findings suggest that ARHL patients may have a higher level of neuroinflammation than age-matched, healthy individuals. Furthermore, validation analysis showed that only those individuals with moderate and moderately severe hearing loss had significantly higher CPV values than HC. This suggests that structural changes in the choroid plexus may occur after a certain level of hearing loss has occurred. The mechanisms behind the increase in CPV associated with ARHL are still unclear, but previous studies have shown that higher CPV in Alzheimer’s patients correlates with greater amyloid-beta deposition and worse cognitive function [25]. Similarly, the volume of EPVS was significantly higher in the ARHL group compared to HC suggesting that the inflow of cerebrospinal fluid into the brain parenchyma is impaired. AQP4, which is polarized in astrocytes, plays a significant role in volume regulation; inflammation can increase its expression which could lead to drainage disorders manifested as enlarged PVS visible on MRI [19].

In addition to structural alternations, our patients with ARHL exhibited lower DTI-ALPS index values compared to HCs. Decreases in ALPS is correlated with declines in several functional measures regarded as a biomarker associated with AD [66]. Our results are also consistent with recent results showing significantly lower DTI-ALPS index values in ARHL patients with cognitive decline versus those with normal cognitive function or normal HCs [32]. Compared to previous study, we combined structural MRI metrics (i.e. CPV and EPVS) to more comprehensively reflect the glymphatic function and also measured the inflammatory factors to understand the changes in glymphatic function at a mechanistic level in older adults with hearing loss. Furthermore, our validation analysis also demonstrated pairwise significant differences of DTI-ALPS index scores among subjects with normal hearing versus those with mild, moderate, and moderately severe hearing loss (except the pairwise comparison of normal vs mild hearing loss and moderate vs moderately severe hearing loss). These results are consistent with differences in inflammation levels between these groups as shown in Supplementary Fig. 2. Our validation analysis showed that as DTI-ALPS index values decreased inflammation and hearing loss increased, consistent with the correlation analyses between hearing ability and each index. In summary, our findings suggest a potential link between glymphatic dysfunction and ARHL. These insights contribute to a better understanding of the underlying mechanisms between hearing loss and cognitive decline in the elderly.

### Inflammatory factors and glymphatic function mediated the association between hearing ability and cognitive function in the elderly

We also explored the role of systemic inflammation and glymphatic dysfunction in mediating the relationship between hearing loss and cognitive decline in older adults. Although mediation analysis cannot provide conclusive causal relationships, it can provide insights for exploring relationships in cross-sectional studies. Previous studies used this approach to determine if loneliness enhanced the likelihood of hearing loss contributing to dementia; however, no significant effect was found [67]. Mediation analysis of cortical morphology revealed that the gyrification index in right cortical area 52 mediated the relationship between hearing ability and executive function [68]. Another mediation analysis reported that subject with worse pure tone thresholds and word-in-noise perception shows greater evidence of brain aging, changes associated poorer cognitive function [69]. The results of our correlation analyses showed that the degree of hearing loss was associated with higher CPV and EPVS metrics, a lower ALPS index, and also correlated with higher levels of inflammation. Furthermore, the ALPS index showed a positive correlation with MoCA scores, while CPV values were negatively correlated with DSST scores, which aligns with prior research linking glymphatic dysfunction to cognitive impairment [25, 32].

Our mediation analysis of glymphatic function and inflammatory factors uncovered novel insights relevant to ARHL. Our analysis revealed that TNF-α mediated the relationship between PTA and ALPS as well as IL-6 mediated the relationship between PTA and ALPS. In addition, ALPS was found to mediate the relationship between PTA and MoCA while CPV was shown to mediate the relationship between PTA and DSST. Subsequent serial mediation analysis revealed that IL-6 and CPV are plausible intermediate factors in the pathway from PTA to DSST. Taken together, these results suggest that increased inflammation and greater dysfunctions in the glymphatic system are collectively contribute to the cognitive decline associated with ARHL. Although mediation analysis suggested indirect links, we recognize that the directionality of these effects cannot be definitively established due to the cross-sectional nature of the data. The mediators were selected based on biological plausibility: IL-6 and TNF-α represent systemic inflammation, while the ALPS index and CPV reflect CSF transport and neuroimmune interface, respectively. This interpretation is supported by a previous study that showed that aging was associated with a dramatic decline in the efficiency of CSF–ISF fluid exchange and interstitial solute clearance and proposed that impaired glymphatic clearance contributes to cognitive decline among the elderly [70]. Our findings suggest that ‘hearing loss—inflammation—glymphatic dysfunction—cognition’ may represent a potential associative pathway. Mediation analysis, though not causal, supports the presence of such indirect links. Future longitudinal or interventional studies are needed to validate the directionality and causality of this mechanism. While this is an intriguing hypothesis, further mechanistic studies are needed to determine how the inflammation induced by ARHL brings about a decline in fluid exchange within the proposed pathway.

Inflammation is regarded as a significant factor in the common-cause hypothesis of cognitive decline in the elderly with hearing loss, consistent with our result linking inflammation, disruption of the glymphatic system and ARHL to cognitive decline. ARHL shares common mechanisms with other age-related diseases in particular immunosenescence and inflamm-aging [11, 71]. As age-related hearing loss progresses, activated microglia release inflammatory factors that disrupt the blood–brain barrier, recruit peripheral macrophages into the central nervous system, and directly induce neuronal degeneration, with complement pathway activation potentially further intensifying neuroinflammation [17]. These changes are also systemic, and immune cells in the nervous system, e.g., microglia and astrocytes, are involved in this process, releasing inflammatory factors that impair glymphatic function, leading to the accumulation of harmful proteins in the brain parenchyma that ultimately results in cognitive decline.

### Limitation

First, one limitation of this study is that it is based on cross-sectional analyses that make it difficult to establish a strong cause and effect relationships. Mediation analysis, while helpful in identifying indirect statistical relationships, should be interpreted cautiously and validated in future longitudinal or interventional studies. Alternative interpretations, such as reverse causality—whereby cognitive impairment leads to reduced engagement, inflammation, or glymphatic dysfunction—are plausible. To validate and extend our findings, future research should incorporate longitudinal cohort studies to assess temporal dynamics and potential causality in the hearing–glymphatic–cognition pathway. Interventional studies evaluating auditory rehabilitation (e.g., hearing aids or cochlear implants) could explore whether restoring auditory input mitigates glymphatic or inflammatory dysfunction. In parallel, experimental models (e.g., aged rodents with induced hearing loss) may provide mechanistic insights into how peripheral sensory deficits interact with central interstitial fluid homeostasis and cognitive circuits. Second, although our study balanced age, gender, years of education, we did not include cardiovascular risk factors, diabetes and hyperlipidemia, medical conditions previously linked to dementia [72–74]. Although we addressed these potential confounders through exclusion criteria, residual confounding from subclinical vascular pathology, sleep quality, or physical frailty may still exist and warrants consideration in future studies. Future studies need to consider these and other possible risk factors that could contribute to dementia in the elderly and consider other neuroinflammatory markers that contribute to cognitive decline. Third, our protocol did not include dynamic contrast-enhanced MRI, aqueduct flow imaging, or phase-contrast CSF techniques, largely due to ethical concerns about contrast agents and technical limitations in older adults. These advanced modalities could provide more direct assessments of CSF dynamics but require further validation in aging populations. Fourth, all participants were older adults of Han Chinese ethnicity recruited from a single-center hospital-based cohort. While this enhances internal consistency, it limits the generalizability of our findings. Ethnic and geographic differences in vascular burden, brain structure, immune response, and auditory health may influence the associations observed. Future research in multi-ethnic and community-based populations is needed to validate these findings.

## Conclusion

By utilizing multimodal MRI metrics, this study has provided insights into the pathological mechanisms linking ARHL to cognition decline, particularly the contribution of chronic inflammation to glymphatic dysfunction and their combined contribution to cognitive decline. In conclusion, our findings shed new light on the mechanism of “hearing loss – inflammation—glymphatic dysfunction—cognition” and suggest that preserving glymphatic function is critical in preventing cognitive decline in older adults with hearing loss. Pharmacotherapies aimed at preventing hearing loss and suppressing chronic inflammation may offer a promising strategy to protect glymphatic function and mitigate cognitive decline [75–78].

## Supplementary Information

Below is the link to the electronic supplementary material.

Supplementary file1 (DOCX 1.02 MB)

## Data Availability

Data generated or analyzed during the study are available from the corresponding author on reasonable request.

## Abbreviations

ARHL: Age-related hearing loss
PTA: Pure tone audiometry
MoCA: Montreal Cognitive Assessment
TNF-α: Tumor necrosis factor
IL-1β: Interleukin-1β
IL-6: Interleukin-6
CPV: Choroid plexus volume
EPVS: Enlarged perivascular space
DTI-ALPS or ALPS: Diffusion tensor image analysis along the perivascular space
