# Supplementary material for: Effect of hearing ability on inflammation and glymphatic function affecting cognition in older adults
Source: GeroScience. 2025 Sep 11;48(1):101–20. doi: 10.1007/s11357-025-01880-7 (PMC12972350; doi:10.1007/s11357-025-01880-7)
Supplement: Supplementary file 1 — Supplementary file1 (DOCX 1.02 MB) [file 11357_2025_1880_MOESM1_ESM.docx]

**Supplementary Materials**

**Effect of hearing ability on inflammation and glymphatic function affecting cognition in older adults**

Weijie Ye#, Chunhua Xing#, Jun Yao, Xiaomin Xu, Zihuai Fang, Xindao Yin, Richard Salvi, Yu-Chen Chen*, Yuexin Cai*

**Supplementary Method 1**

**Supplementary Table 1**

**Supplementary Table 2**

**Supplementary Table 3**

**Supplementary Table 4**

**Supplementary Table 5**

**Supplementary Figure 1**

**Supplementary Figure 2**

**Supplementary Method 1**

MRI acquisition

All participants wore earplugs and foam padding to mitigate scanner noise and head motion. During scanning, subjects were instructed to keep their eyes closed and stay awake without thinking about anything in particular. Images consist of: (1) high-resolution three-dimensional turbo fast-echo (3D-TFE) T1-weighted sequences (TR = 8.1 ms, TE = 3.7 ms, slices = 170, thickness = 1 mm, gap = 0 mm, FA = 8°, matrix = 256 × 256, FOV = 256 mm × 256 mm); (2) DTI images using a single­shot echo planar imaging (EPI) sequence (TR = 10s, TE = 95 ms, slices = 70, slice thickness = 2 mm, gap = 0 mm, FA = 90°, b-values = 0 and 1000 s/mm^2^, diffusion gradient directions = 30, matrix = 128 × 128, FOV = 256 mm × 256 mm). All images were manually examined by an experienced radiologist who had no knowledge of the participants' personal information.

**Supplementary Table 1.** Significant partial spearman rank correlation between variables including PTA, glymphatic indexes, inflammatory factors, and cognitive performance after Benjamini-Hochberg FDR correction

| Variable1 | Variable2 | Spearman r | *p* value | *FDR q* |
| --- | --- | --- | --- | --- |
| MoCA | PTA | -0.218388406 | 0.000532776 | 0.004348 |
| CFTd | PTA | -0.225863495 | 0.000336596 | 0.002937 |
| CPV | PTA | 0.193058352 | 0.002260337 | 0.013948 |
| ALPS | PTA | -0.334230547 | 6.93837E-08 | <0.0001 |
| EPVS | PTA | 0.231142801 | 0.000241137 | 0.00226 |
| TNF-α | PTA | 0.760685915 | 4.52406E-48 | <0.0001 |
| IL-6 | PTA | 0.751350648 | 2.63622E-46 | <0.0001 |
| IL-1β | PTA | 0.76273536 | 1.80752E-48 | <0.0001 |
| ALPS | MoCA | 0.174833016 | 0.005768508 | 0.03394 |
| TNF-α | MoCA | -0.226200073 | 0.000329589 | 0.002978 |
| IL-6 | MoCA | -0.196849638 | 0.0018403 | 0.012584 |
| IL-1β | MoCA | -0.231681731 | 0.000232965 | 0.002267 |
| CDT | VFT | 0.195055312 | 0.002029301 | 0.013511 |
| TNF-α | TMTB | 0.174473359 | 0.005871189 | 0.033759 |
| CPV | DSST | -0.19172674 | 0.002427445 | 0.014622 |
| AVLTd | DST | 0.197907079 | 0.001736589 | 0.012204 |
| AVLTd | AVLT | 0.815920144 | 1.81209E-60 | <0.0001 |
| TNF-α | CFTd | -0.217151635 | 0.000573996 | 0.004401 |
| IL-6 | CFTd | -0.270378067 | 1.58103E-05 | 0.0002 |
| IL-1β | CFTd | -0.250364706 | 6.70543E-05 | 0.000771 |
| GMV | TIV | 0.787160522 | 1.49774E-53 | <0.0001 |
| WMV | TIV | 0.826339276 | 2.79177E-63 | <0.0001 |
| CSF | TIV | 0.699371649 | 9.60869E-38 | <0.0001 |
| EPVS | TIV | -0.235478693 | 0.000182308 | 0.001845 |
| WMV | GMV | 0.815838656 | 1.90312E-60 | <0.0001 |
| CSF | GMV | 0.193831123 | 0.002168231 | 0.013714 |
| CPV | GMV | -0.172993193 | 0.006311219 | 0.035483 |
| EPVS | GMV | -0.358909296 | 5.93522E-09 | <0.0001 |
| CSF | WMV | 0.280773711 | 7.12445E-06 | <0.0001 |
| EPVS | WMV | -0.303547981 | 1.10721E-06 | <0.0001 |
| CPV | CSF | 0.296441386 | 2.01391E-06 | <0.0001 |
| TNF-α | CSF | 0.167085371 | 0.008375891 | 0.046067 |
| IL-6 | CSF | 0.246245503 | 8.89911E-05 | 0.000938 |
| EPVS | CPV | 0.222388045 | 0.000417497 | 0.003521 |
| TNF-α | CPV | 0.194752982 | 0.002062835 | 0.013382 |
| IL-6 | CPV | 0.248366145 | 7.6971E-05 | 0.000847 |
| IL-1β | CPV | 0.217223149 | 0.000571534 | 0.004519 |
| TNF-α | ALPS | -0.369926724 | 1.84657E-09 | <0.0001 |
| IL-6 | ALPS | -0.29336725 | 2.59595E-06 | <0.0001 |
| IL-1β | ALPS | -0.304364995 | 1.03256E-06 | <0.0001 |
| TNF-α | EPVS | 0.254343738 | 5.07796E-05 | 0.000612 |
| IL-6 | EPVS | 0.210317971 | 0.000860039 | 0.006217 |
| IL-1β | EPVS | 0.213867612 | 0.000698197 | 0.005195 |
| IL-6 | TNF-α | 0.757363301 | 1.9636E-47 | <0.0001 |
| IL-1β | TNF-α | 0.752539814 | 1.58664E-46 | <0.0001 |
| IL-1β | IL-6 | 0.749009568 | 7.1032E-46 | <0.0001 |

Abbreviations: PTA, pure tone audiometry; MoCA, Montreal Cognitive Assessment; VFT, Verbal Fluency Test; TMT-B, Trail Making Test-Part B; DSST, Digit Symbol Substitution Test; DST, Digit Span Test; AVLT, Auditory Verbal Learning Test; AVLTd, Auditory Verbal Learning Test-delayed; CDT, Clock Drawing Test; CFTd, Complex Figure Test-delayed; TIV, total intracranial volume; GMV, gray matter volume; WMV, white matter volume; CSF, cerebrospinal fluid; CPV, choroid plexus volume; EPVS, enlarged perivascular space; DTI-ALPS, diffusion tensor image analysis along the perivascular space, also abbreviated as ALPS. FDR q, corrected for multiple comparisons using Benjamini-Hochberg method.

**Supplementary Table 2.** Simple Mediation analysis of inflammatory factors between hearing and glymphatic function

|  | | X: PTA M: TNF-α Y: ALPS | | | | | | |
| --- | --- | --- | --- | --- | --- | --- | --- | --- |
| Model | ===> | | Outcome | Coefficient | Std coefficient | *p* | LLCI | ULCI |
| PTA | ===> | | TNF-α | 1.5985 | 0.8749 | **<0.0001** | 1.4881 | 1.7090 |
| TNF-α | ===> | | ALPS | -0.0057 | -0.4521 | **0.0002** | -0.0087 | -0.0028 |
| PTA |  |  |  | 0.0007 | 0.0294 | 0.8041 | -0.0047 | 0.0061 |
| Total effect | | | | -0.0085 | -0.3661 | **<0.0001** | -0.0111 | -0.0058 |
| Direct effect | | | | 0.0007 | 0.0294 | 0.8041 | -0.0047 | 0.0061 |
| Indirect effect | | | | -0.0091 | -0.3955 | / | -0.0144 | -0.0040 |
|  | | X: PTA M: IL-6 Y: CPV | | | | | | |
| Model | ===> | | Outcome | Coefficient | Std coefficient | *p* | LLCI | ULCI |
| PTA | ===> | | IL-6 | 0.3495 | 0.8450 | **<0.0001** | 0.3219 | 0.3772 |
| IL-6 | ===> | | CPV | 0.0171 | 0.2363 | **0.0414** | 0.0007 | 0.0336 |
| PTA |  |  |  | 0.0002 | 0.0052 | 0.9637 | -0.0067 | 0.0070 |
| Total effect | | | | 0.0062 | 0.2049 | **0.0011** | 0.0025 | 0.0098 |
| Direct effect | | | | 0.0002 | 0.0052 | 0.9637 | -0.0067 | 0.0070 |
| Indirect effect | | | | 0.0060 | 0.1997 | / | 0.0001 | 0.0117 |

PTA, pure tone audiometry; IL-6, interleukin-6; TNF-α, tumor necrosis factor; CPV, choroid plexus volume; DTI-ALPS, diffusion tensor image analysis along the perivascular space, also abbreviated as ALPS.

Bold values represent significant differences.

**Supplementary Table 3.** Simple Mediation analysis of glymphatic function between hearing and cognitive

|  | | X: PTA M: ALPS Y: MoCA | | | | | | |
| --- | --- | --- | --- | --- | --- | --- | --- | --- |
| Model | ===> | | Outcome | Coefficient | Std coefficient | *p* | LLCI | ULCI |
| PTA | ===> | | ALPS | -0.0085 | -0.3661 | **<0.0001** | -0.0111 | -0.0058 |
| ALPS | ===> | | MoCA | 0.6292 | 0.1318 | **0.0467** | 0.0092 | 1.2491 |
| PTA |  |  |  | -0.0196 | -0.1780 | 0.0074 | -0.0339 | -0.0053 |
| Total effect | | | | -0.0249 | -0.2263 | 0.0003 | -0.0383 | -0.0115 |
| Direct effect | | | | -0.0196 | -0.1780 | 0.0074 | -0.0339 | -0.0053 |
| Indirect effect | | | | -0.0053 | -0.0482 | / | -0.0115 | -0.0001 |
|  | | X: PTA M: CPV Y: DSST | | | | | | |
| Model | ===> | | Outcome | Coefficient | Std coefficient | *p* | LLCI | ULCI |
| PTA | ===> | | CPV | 0.0062 | 0.2049 | **0.0011** | 0.0025 | 0.0098 |
| CPV | ===> | | DSST | -0.7809 | -0.2126 | **0.0009** | -1.2398 | -0.3219 |
| PTA |  |  |  | 0.0036 | 0.0330 | 0.6039 | -0.0101 | 0.0174 |
| Total effect | | | | -0.0012 | -0.0106 | 0.8671 | -0.0149 | 0.0126 |
| Direct effect | | | | 0.0036 | 0.0330 | 0.6039 | -0.0101 | 0.0174 |
| Indirect effect | | | | -0.0048 | -0.0436 | / | -0.0094 | -0.0013 |

PTA, pure tone audiometry; MoCA, Montreal Cognitive Assessment; DSST, Digit Symbol Substitution Test; CPV, choroid plexus volume; DTI-ALPS, diffusion tensor image analysis along the perivascular space, also abbreviated as ALPS.

Bold values represent significant differences.

**Supplementary Table 4.** Serial Mediation analysis of inflammatory factors and glymphatic function between hearing and cognition

|  | | X: PTA M1: IL-6 M2: CPV Y: DSST | | | | | | |
| --- | --- | --- | --- | --- | --- | --- | --- | --- |
| Model | ===> | | Outcome | Coefficient | Std coefficient | *p* | LLCI | ULCI |
| PTA | ===> | | IL-6 | 0.3495 | 0.8450 | **<0.0001** | 0.3219 | 0.3772 |
| IL-6 | ===> | | CPV | 0.0171 | 0.2363 | **0.0414** | 0.0007 | 0.0336 |
| PTA |  |  |  | 0.0002 | 0.0052 | 0.9637 | -0.0067 | 0.0070 |
| CPV | ===> | | DSST | -0.8076 | -0.2199 | **0.0007** | -1.2706 | -0.3446 |
| IL-6 |  |  |  | 0.0275 | 0.1031 | 0.3798 | -0.0340 | 0.0890 |
| PTA |  |  |  | -0.0058 | -0.0527 | 0.6508 | -0.0310 | 0.0194 |
| Total effect | | | | -0.0012 | -0.0106 | 0.8671 | -0.0149 | 0.0126 |
| Direct effect | | | | -0.0058 | -0.0527 | 0.6508 | -0.0310 | 0.0194 |
| Indirect effect  PTA => IL-6 => DSST | | | | 0.0096 | 0.0871 | / | -0.0129 | 0.0314 |
| Indirect effect  PTA => CPV => DSST | | | | -0.0001 | -0.0012 | / | -0.0056 | 0.0052 |
| Indirect effect  PTA => IL-6 => CPV => DSST | | | | -0.0048 | -0.0439 | / | -0.0109 | -0.0002 |

PTA, pure tone audiometry; IL-6, interleukin-6; CPV, choroid plexus volume; DSST, Digit Symbol Substitution Test.

Bold values represent significant differences.

**Supplementary Table 5.** Demographic and characteristics of participants grouped by high frequency PTA

| Variable | Normal  (n=77) | Mild  (n=54) | Moderate  (n=48) | Moderately severe  (n=72) | Test statistic | *p* value |
| --- | --- | --- | --- | --- | --- | --- |
| Age, years | 63.66 (3.87) | 64.00 (3.50) | 64.67 (3.20) | 63.33 (3.09) | 6.178 | 0.103 |
| Gender, F/M | 32/45 | 28/26 | 23/25 | 33/39 | 1.422 | 0.700 |
| Education, years | 11.29 (2.03) | 11.63 (2.15) | 11.06 (1.88) | 11.93 (2.21) | 5.557 | 0.135 |
| Mean PTA at 4 kHz, dB HL | 16.92 (4.11) | 24.12 (6.30) | 42.92 (5.34) | 53.13 (4.92) | 213.244 | **<0.0001** |
| Mean PTA at 8 kHz, dB HL | 16.53 (5.04) | 26.62 (11.71) | 53.54 (5.36) | 61.56 (5.81) | 201.615 | **<0.0001** |
| **Neuropsychological assessments** | | | | | | |
| MMSE | 28.86 (1.18) | 29.19 (0.99) | 28.96 (1.01) | 28.74 (1.01) | 7.835 | **0.050** |
| MoCA | 27.21 (1.20) | 27.33 (1.36) | 26.85 (2.01) | 26.29 (1.98) | 13.864 | **0.003** |
| VFT | 14.38 (3.38) | 14.55 (3.08) | 14.45 (3.53) | 14.26 (4.03) | 0.518 | 0.915 |
| TMT-A, second | 67.65 (17.81) | 62.30 (14.30) | 77.19 (29.02) | 71.56 (26.21) | 7.118 | 0.068 |
| TMT-B, second | 180.75 (52.43) | 174.72 (55.35) | 199.60 (54.04) | 190.85 (54.92) | 6.447 | 0.092 |
| DSST | 69.99 (9.47) | 69.04 (7.74) | 68.71 (7.46) | 70.01 (9.27) | 0.881 | 0.830 |
| DST | 11.57 (1.86) | 11.65 (1.80) | 11.54 (2.00) | 11.07 (1.68) | 3.646 | 0.302 |
| AVLT | 35.49 (6.97) | 35.48 (6.63) | 34.96 (8.37) | 34.93 (7.43) | 0.429 | 0.934 |
| AVLTd | 6.73 (2.45) | 6.83 (2.33) | 6.90 (2.55) | 7.13 (2.29) | 1.217 | 0.749 |
| CDT | 3.45 (0.57) | 3.59 (0.50) | 3.50 (0.62) | 3.57 (0.50) | 2.007 | 0.571 |
| CFT | 34.32 (1.87) | 34.28 (2.72) | 31.94 (5.78) | 32.31 (6.35) | 7.734 | 0.052 |
| CFTd | 21.32 (5.71) | 21.02 (5.32) | 18.78 (4.72) | 17.75 (4.48) | 21.464 | **<0.0001** |
| **Inflammatory factors** | | | | | | |
| TNF-α | 13.70 (2.48) | 18.92 (11.81) | 42.65 (7.88) | 45.55 (9.02) | 168.389 | **<0.0001** |
| IL-1β | 9.81 (1.14) | 13.49 (8.75) | 32.83 (9.69) | 32.65 (9.18) | 167.617 | **<0.0001** |
| IL-6 | 3.32 (1.53) | 4.56 (2.96) | 10.35 (1.69) | 10.01 (1.64) | 168.364 | **<0.0001** |
| **MRI indexes** | | | | | | |
| TIV, cm^3^ | 1370.33 (149.99) | 1333.12 (138.26) | 1360.83 (164.37) | 1387.83 (145.21) | 5.081 | 0.166 |
| GMV, cm^3^ | 589.40 (50.70) | 569.43 (49.23) | 570.33 (58.78) | 577.12 (62.89) | 5.289 | 0.152 |
| WMV, cm^3^ | 501.05 (52.39) | 486.78 (56.05) | 491.79 (64.43) | 499.31 (63.34) | 3.225 | 0.358 |
| CSF, cm^3^ | 279.88 (81.59) | 276.91 (64.77) | 298.71 (93.09) | 311.40 (81.58) | 8.652 | **0.034** |
| CPV, cm^3^ | 1.83 (0.43) | 1.90 (0.44) | 2.01 (0.45) | 2.06 (0.41) | 10.560 | **0.014** |
| CPV,  ratio of TIV*10^3^ | 1.33 (0.27) | 1.43 (0.30) | 1.47 (0.26) | 1.48 (0.25) | 14.378 | **0.002** |
| EPVS, cm^3^ | 2.13 (0.50) | 2.15 (0.57) | 2.36 (0.51) | 2.35 (0.58) | 19.095 | **0.0003** |
| EPVS,  ratio of TIV*10^3^ | 1.56 (0.33) | 1.61 (0.36) | 1.75 (0.37) | 1.70 (0.40) | 11.837 | **0.008** |
| DTI-ALPS | 1.63 (0.21) | 1.61 (0.18) | 1.47 (0.18) | 1.47 (0.21) | 32.389 | **<0.0001** |

Abbreviations: F, female; M, male; PTA, pure tone audiometry; MMSE, Minimum Mental State Examination; MoCA, Montreal Cognitive Assessment; VFT, Verbal Fluency Test; TMT-A, Trail Making Test-Part A; TMT-B, Trail Making Test-Part B; DSST, Digit Symbol Substitution Test; DST, Digit Span Test; AVLT, Auditory Verbal Learning Test; AVLTd, Auditory Verbal Learning Test-delayed; CDT, Clock Drawing Test; CFT, Complex Figure Test; CFTd, Complex Figure Test-delayed; TNF-α, tumor necrosis factor; IL-1β, interleukin-1β; IL-6, interleukin-6; TIV, total intracranial volume; GMV, gray matter volume; WMV, white matter volume; CSF, cerebrospinal fluid; CPV, choroid plexus volume; EPVS, enlarged perivascular space; DTI-ALPS, diffusion tensor image analysis along the perivascular space, also abbreviated as ALPS.

Values are presented as mean (1SD). Kruskal-Wallis test was used for comparison between multiple groups. Bold values represent significant differences.


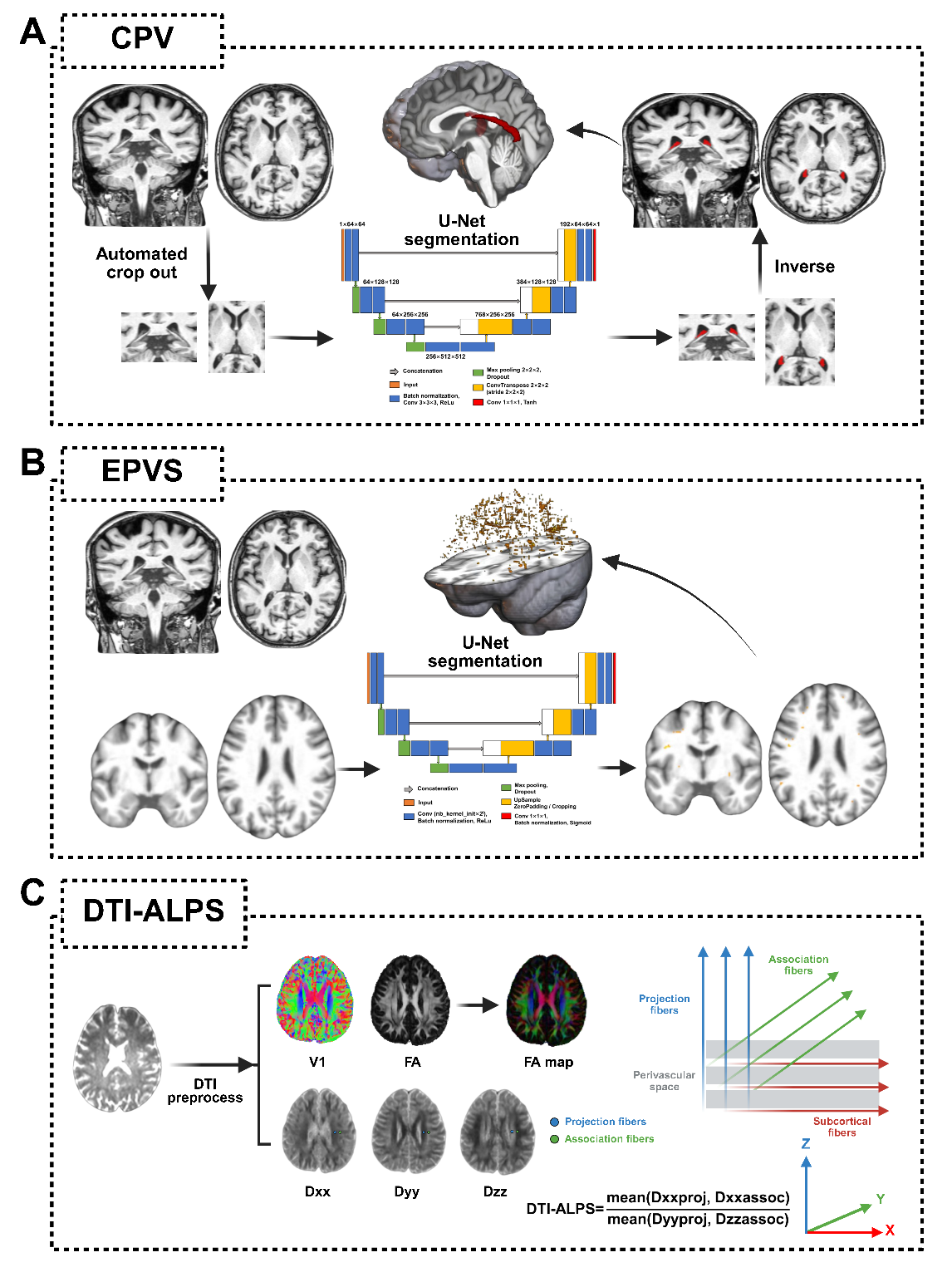


**Supplementary Figure 1.** The MRI indices calculation flow of glymphatic system function. (A) Choroid plexus was segmented by inputting T1w image into a validated U-net deep learning model, and its volume was extracted after inspected and reviewed. (B) EPVS probability maps were obtained by using another validated U-net deep learning segmentation model. The volume of EPVS is extracted after thresholding and review. (C) After preprocessing of DTI, FA and individual diffusivity maps were generated using DTIFIT tool implemented in FMRIB Software Library. Next, placing 5mm diameter spherical ROIs on the bilateral projection fibers, association fibers, and subcortical fibers and extracting the diffusivities of the three directions along the x, y, and z axes at the ROIs on bilateral fibers on both sides of the brain. The DTI-ALPS index was calculated based on this formula: mean (Dxxassoc, Dxxproj) / mean (Dzzassoc, Dyyproj). Abbreviations: EPVS, enlarged perivascular space; DTI, diffusion tensor image; FA, fractional anisotropy; ROI, regions of interest. Created with BioRender.com


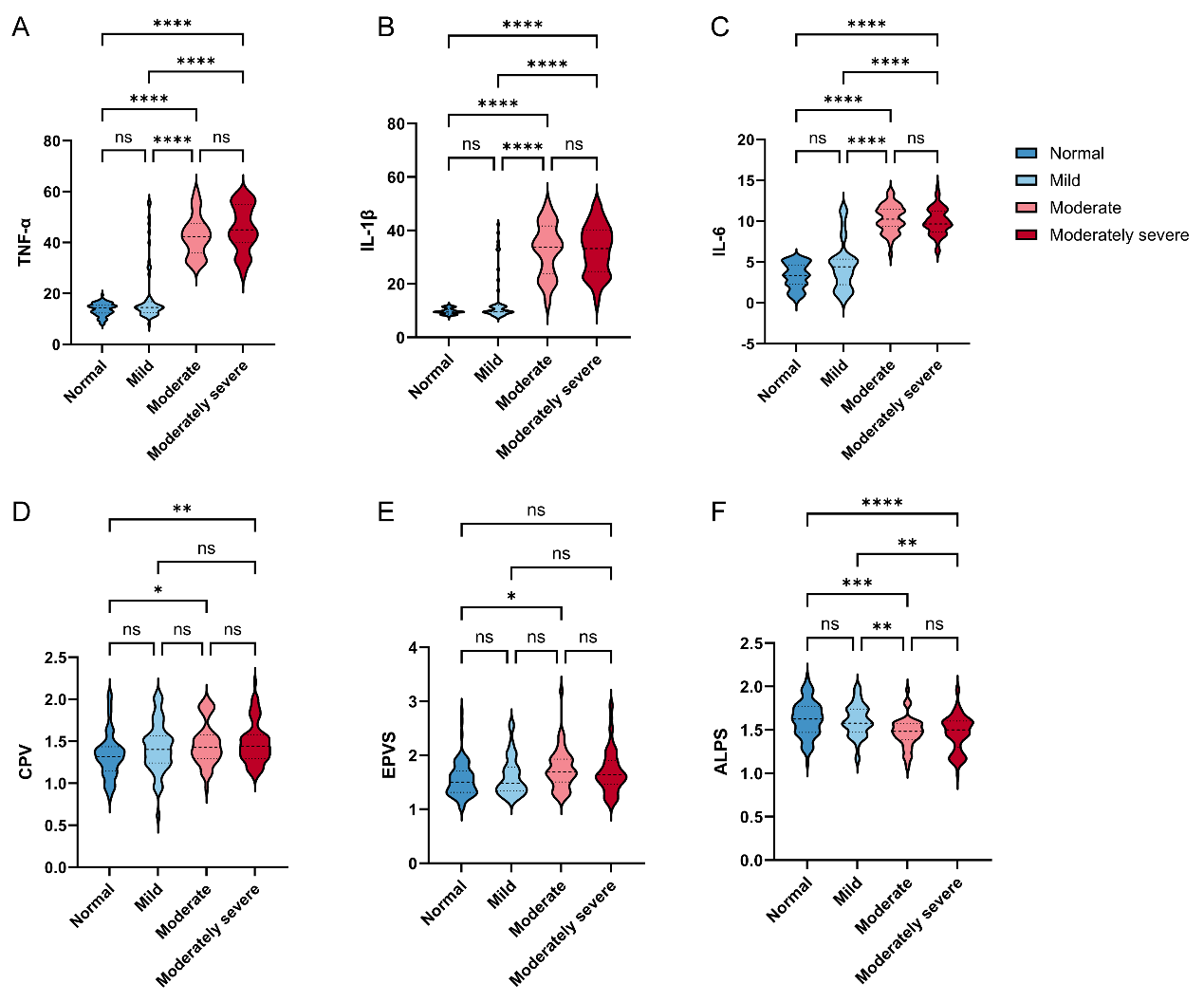


**Supplementary Figure 2.** Validation analysis results of regrouping based on the average PTA at 4 kHz and 8 kHz in the better ear. Violin plots show pairwise comparisons of TNF-α (A), IL-1β (B), IL-6 (C). There were significant differences in group comparisons of normal versus moderate, normal versus moderately severe, mild versus moderate, and mild versus moderately severe. (D) CPV showed significant difference in comparisons of normal versus moderate, and normal versus moderately severe. (E) EPVS showed significant difference in comparisons of normal versus moderate. (F) DTI-ALPS showed significant difference in comparisons of normal versus moderate, normal versus moderately severe, mild versus moderate, and mild versus moderately severe.

Groups were compared using the Dunn’s post hoc test for multiple comparison correction. The asterisks (*, **, ***, and ****) indicate statistical significance at *p <* 0.05, *p <* 0.01, *p <* 0.001, and *p* < 0.0001, respectively. Abbreviations: ns, no significant; TNF-α, tumor necrosis factor; IL-1β, interleukin-1β; IL-6, interleukin-6; CPV, choroid plexus volume; EPVS, enlarged perivascular space; DTI-ALPS, diffusion tensor image analysis along the perivascular space, also abbreviated as ALPS.
